# Academy of Stomatology

**Published:** 1905-04

**Authors:** 

**Affiliations:** Academy of Stomatology


### Reports of Society Meetings.

### ACADEMY OF STOMATOLOGY.

A regular meeting of the Academy of Stomatology of Philadelphia was held at its rooms, 1731 Chestnut Street, on the evening of Tuesday, October 25, 1904, the President, Dr. I. N. Broomell, in the chair.

Dr. A. H. Macpherson read a paper entitled "Electrolysis for the Treatment of Peridental Inflammations."

(For Dr. Macpherson's paper, see page 32.)

### DISCUSSION.

*Dr. Albert P. Brubaker.*—The statements made by Dr. Macpherson regarding the treatment of abscesses with the electric current are most interesting and instructive. An explanation of the facts presented is to be found in what is known of the effects of the passage of an electric current through solutions of inorganic salts, organic matter, and upon lower organisms.

With the passage of the electric current through a solution of any inorganic salt, for example, sodium chloride, the salt is decomposed, the chlorine passing to the positive pole, or anode, the sodium passing to the negative pole, or cathode.

If the current is made to flow through a more or less coagulated mass of albumin, it will be found after a short time that the albumin in the neighborhood of the anode is coagulated still more, while in the neighborhood of the cathode it is liquefied. The intermediate portion may or may not be affected, according to the strength of the current. If the current is made to flow through a small cell containing low forms of animal life, they will arrange themselves along certain lines and begin to move toward the negative pole or cathode.

These facts may be utilized in explanation of the facts presented by Dr. Macpherson. Thus an abscess around a tooth, or elsewhere, is a cavity containing pus, which consists of water, inorganic salts, albumin, white blood-curpuscles, pus-cells, and micro-organisms. The tissue overlying the abscess consists largely of albumin. If the electric current enters the body at any indifferent point—*e.g.*, the hand, the back of the neck, or elsewhere—through a small electrode, the tissues at that point will be more or less coagulated. As it leaves through the cathode the tissues will be liquefied. In the method of treatment advocated by Dr. Macpherson a small electrode, the cathode, is placed on the outer surface of the gum overlying the abscess and the current established. On entering the body the current at once diffuses and takes the lines of least resistance, but as it approaches the cathode the lines again converge. As it passes through the abscess contents and the overlying tissues, the inorganic salts are decomposed, pus-cells and bacteria accumulate in the neighborhood of the cathode, and the albumin of the overlying tissues is liquefied. The continuous flow of the current soon establishes one or more outlets, after which the contents of the cavity are discharged on the surface. With this event the swelling, pressure, and pain subside and repair is initiated. Similar effects are produced, no doubt, in the walls of the cavity.

In this electrolytic treatment the coagulation which would occur at the anode is prevented by the use of a large sponge electrode, so that the current will enter the body through a large area of surface. It is not the quantity, but the density, of the current which coagulates the tissues, which at this point is not desirable; but at the cathode, where electrolytic effects are desirable, the electrode should be small, as in the apparatus employed by Dr. Macpherson. At this point density of current is required.

*Mr. F. Geiger.*—I do not think I can add anything to what Drs. Macpherson and Brubaker have said. I have my battery here with the various electrodes, and am prepared to give a demonstration of the work, if we have a patient.

In treating an abscess or a boil that is just forming, if electrodes are properly applied it will bring the pus to the surface in a few moments without any breaking of the skin.

*Dr. J. H. Gaskill.*—I would like to ask what effect there is on healthy tissue?

*Mr. Geiger.*—There is no effect at all upon healthy tissue. Where there is pus, if you apply the positive pole to the gum you can drive the pus out into the system if you leave it on long enough. I have taken the pus from one arm to another.

*Dr. J. Head.*—How do you know you did not draw the pus from other parts of the body?

*Mr. Geiger.*—In a healthy body an electrode left on all day will not draw inflammation to a point; but if there is pus in the path of the current it will come out at the negative electrode. I can draw pus from one place to another.

*Dr. M. H. Cryer.*—We employ the same process for tumors, except that we introduce long needles, and if we want to draw out, we use the negative pole; if to drive in, we use the positive.

*Dr. Macpherson.*—I had a patient who was unable to open his mouth for three days, caused by an impacted lower wisdom-tooth. After the application of electrolysis there was immediate relaxation of the muscles, and there was even more relaxation after a second application. The applications were from fifteen to eighteen minutes' duration, with twenty minutes' interval. The negative pole was placed immediately over the wisdom-tooth and the positive pole at the back of the neck. I also had a patient, a dentist, suffering from an abscess on a left upper bicuspid which she had not been able to make discharge. Pus was discharged upon the application of the electrode by pressure, and on this account the case was not a good one to prove the power of electrolysis. There was, however, relief from the pain, and the swelling decreased. The next day I applied the lancet, and some pus which had accumulated came out. Mr. Geiger said I did not apply the electricity sufficiently long. I never saw an abscess heal so quickly. In two days the gum was in a perfectly normal condition, and now, after two weeks, it remains the same.

*Dr. Albert P. Brubaker.*—I should like to call the attention of the society to a statement made some years ago, which did not receive very much consideration at the time. It was at the close of a short paper on the causation of dental erosion. It was stated that, as in many cases the mouths of glands on the posterior surface of the upper lip are enlarged, swollen, and discharging an acid fluid, which it is believed is the cause of the erosion, the electric current might be employed for the destruction of these glands and the progress of the erosion checked. The method would be similar to that employed by electro-surgeons for the removal of superfluous hairs. A large electrode, the anode, could be placed on the hand and a needle electrode, the cathode, inserted into the mouth of the gland. In a few minutes the gland structures would be destroyed. If but a dozen of the glands are diseased, it would not be difficult or tedious to destroy them and thus check the further progress of the erosion.

*Dr. James Truman.*—I am not familiar with this procedure, therefore I can say nothing about it. I was much pleased, however, both in hearing the paper and with Dr. Brubaker's lucid explanation of the process.

*Dr. M. I. Schamberg.*—We are all very anxious to ascertain just what can be accomplished in dentistry by this new method of treatment. As was said, electrolysis has been practised in medicine for some time, but its mode of application to medicine differs somewhat from the method advocated this evening. I can conceive of the possibility of the destruction of tissue by the production of an electric burn at the point where the negative pole is placed; but to draw pus through the tissues for some distance is a phenomenon with which I am not familiar. It is possible that it may have been done, as is evidenced by a very favorable report of the clinic given by Dr. Macpherson at the State convention this year. I am sorry that I did not see the demonstration, for I have a suspicion that pressure upon the abscessed area caused the pus to exude. This suspicion is somewhat increased by Dr. Macpherson's report of this evening, in which he refers to the case in which he practically admitted that pressure brought the pus through an existing fistula at the first application. It is not unreasonable to suppose that if the fistula existed during the first application, it had not entirely healed when a second application was made. I am taking a sceptical view of this question merely to bring out the truth of the matter. If this method of treatment is to be helpful, we all want to adopt it. If it falls short of doing that which is claimed for it, we naturally want to waste little time with it. I think it is a mistake to allude to electrolysis as a cure for alveolar abscess. It may do as much as the knife will do, and in a neater way, without the aspect of a surgical procedure, but no greater claim should be made than that it will evacuate pus, for when we speak about curing an alveolar abscess by the mere removal of pus we make a big mistake. Many abscesses recur because of the fact that the evacuation of pus is insufficient treatment. If we wish to permanently cure our cases we must remove the cause, and in a large proportion of cases the cause lies in the necrotic end of a root. Most often the surrounding bone is diseased, and unless the débris is removed from the periapical space permanent relief will not be afforded.

I believe it would be a hazardous procedure to cause pus to be passed from one portion of the body to another, if, as is stated by Mr. Geiger, such can be done, for the entire system might thus become infected. This same danger applies to the gum-tissue and alveolus. It puts upon nature a double duty, that of curing the part already affected and the portion through which the pus is made to pass.

It is disappointing not to hear a report of more cases treated, for the few alluded to by the essayist are not, to my mind, sufficiently conclusive. I shall be only too glad to be convinced of the value of this method if it possesses the virtue ascribed to it.

*Dr. Gaskill.*—I think that Dr. Schamberg's objections in some respects are not very well founded. I have a great deal of faith in the instrument from the results which have been stated. Dr. Schamberg speaks of the possibility of infection by drawing the pus. Whenever pus is evacuated, by whatever means, whether it goes through a cut surface or not, it may cause infection. So, I think that these points are not as well taken as they might have been.

*Dr. Macpherson.*—My first subject was a patient of my neighbor, Dr. Shoemaker. I made the application, gave him a looking-glass, and had him observe the result. There was over a teaspoonful of pus accumulated at the electrode. That was a year ago, and six months afterwards the part was in perfect condition. He had never had an abscess heal with such satisfactory results. The gentleman who brought the patient to the State meeting had been experimenting with radium and with electricity and he did not believe it was possible to get the results claimed. I was uncertain whether to apply the electrode for pain or for pus. If applied for pain, I would move the electrode; if for pus, I would hold it in one position. I tried for pus, and the gentleman was absolutely convinced, and said he thought the result was marvellous. I have had a number of other cases, and I think I can say there have been no failures. I have proceeded with great timidity, and would not have read the paper before the State meeting had I not been urged by Dr. Broomell. I would like to have made more exact records of the work, but the apparatus is ready, and I think it is safe for all to make their own experiments.

*Dr. A. N. Gaylord.*—If this method is what it appears and is claimed to be, it is certainly one of vital importance to every one of us, and the apparatus will find a welcome place in every office. However, I am a little sceptical, and would very much dislike to see the opinion of this meeting to go on record as accepting this method conclusively. Mr. Geiger states that the material which accumulates at the negative electrode is pus, but admits that his belief is not substantiated by bacteriological test.

I would like to ask Dr. Macpherson whether, to his knowledge, any other test has been made except the one which Dr. Broomell made.

*Dr. Broomell.*—The chemical test showed about seventy-five per cent. pus.

*Dr. Gaylord.*—Dr. Broomell tells me that the case from which he made a test was one in which there was an open sinus, therefore, as far as proving anything relative to this method, it is valueless.

The product from a case where it is claimed that pus has been drawn through the tissues should by all means be given a thorough test, and not be accepted as pus because it looks like pus. Many a man has purchased a gold brick because it looked like gold.

I have done a little work in a crude way by adding resistance to the electro-dental switch-board, and while I was gratified with the result, feeling that I gave relief to the patient, yet the accumulation around the negative electrode was not to my mind pus. I believe this product to be the effect of electrolysis on mucus and saliva.

I witnessed the case of which Dr. Macpherson speaks at the meeting of the Pennsylvania State Society, in which case he states that he obtained a teaspoonful of pus. A teaspoonful of pus, if confined within the bone, would mean a very large necrotic area, while if it were within the soft tissues, we would naturally look for considerable swelling. The patient under question had no apparent swelling, and the history would indicate that his trouble had not passed beyond the stage of pericemental inflammation, and yet it is stated that a teaspoonful of pus was extracted. It looks to me, in this case, as if the accumulation must have been something other than pus, although it had that appearance.

I am by no means trying to cry down this method, but I would like to see the conclusions based on scientific investigation. We should not accept this treatment too readily, and risk becoming the laughing stock of the profession in the future.

Electrolysis has been known to the medical profession for many years, and if it is capable of producing such wonderful results, it seems strange to me that they should not have discovered it ere this.

*Dr. Broomell.*—They didn't discover anæsthesia.

*Dr. Gaylord.*—This is very true, but the medical profession has had this apparatus and similar ones manufactured by Mr. Geiger in use for many years, and if it does what he claims for it, it has a much larger field in medicine than in dentistry,—for instance, the venereal specialist in his battle with gonococcus.

*Dr. Head.*—I think the question is not whether it is true or false pus, whether it looks like mucus or saliva, but, Does this current extract the bacteria from the tissues? The whole thing comes down fundamentally to whether that which is extracted from the tissues contains bacteria. It seems to me the scientific way of deciding that point is to extract the fluid from a place that is supposed to contain pus, and then at once examine it under the microscope. If the bacteria are present I should think they surely would appear under those conditions. The question of whether there are white blood-corpuscles is a minor matter. It is hardly credible that the fluid would be purely in the form of pus. It is more probable that there would be a large admixture of blood-serum.

*Dr. Gaylord.*—That is my argument. If the product is blood-serum, call it blood-serum; do not call it pus.

*Dr. Schamberg.*—I would like to ask Dr. Macpherson whether he extracted any material resembling pus from the case in which the patient was suffering from an impacted wisdom-tooth.

*Dr. Macpherson.*—Yes, a considerable amount of pus. The pus as it adheres to the electrode does not look like the thick pus you get from the lancet; it is much thinner than the ordinary pus.

If not pus, what became of the pus that was causing the abscess and which this treatment relieved most thoroughly?

*Dr. Schamberg.*—Did you determine whether the patient acquired the use of his jaw through electrical stimulation, which would be possible if tetanic contraction of the muscles prevailed, or was the muscular relaxation due to the actual withdrawal of pus from about the tooth? Was the relief immediate?

*Dr. Macpherson.*—Yes; as I stated, as soon as the patient was through with the operation, he was on his feet. He said, immediately, "I can open my mouth."

*Dr. Schamberg.*—If that is the case, I believe the effect was due to electrical stimulation rather than to the withdrawal of pus. Even though pus was removed, it is hardly possible that the muscles would have acquired their normal function so promptly had their rigidity been due to inflammatory action produced by the toxic influence of pyogenic organisms. If a mouth-gag had been applied to the patient's jaw and the muscles exercised thereby, it is a question whether mechanical stimulation would not have acquired for the jaw the same amount of motion as was accomplished by electrical stimulation.

*Dr. Macpherson.*—In every case in which electrolysis was used the patient felt decidedly better after the operation. The swelling disappeared and the gum resumed its normal feeling to the patient.

*Dr. J. C. Curry.*—As a matter of history, I would like to say that to my knowledge Dr. Flagg used a method similar to this some ten years ago. He wrote considerably on the subject and gave very interesting demonstrations.

*Mr. Geiger.*—That was Faradism. This is pure galvanism.

*Dr. Schamberg.*—Dr. M. L. Rhein has been using the electric method for curing abscess at the apices of teeth. He does it by passing an electrode through the canal. I believe that many cures have been effected by this method. I must admit that I am still sceptical about the possibility of drawing pus through tissues causing the complete evacuation of pus cavities when there is no fistula present.

*Dr. Broomell.*—There has been sufficient discussion to show that the process has not been absolutely commended at the present time. I am glad that there have been opinions expressed on both sides.

OTTO E. INGLIS,
*Editor Academy of Stomatology.*

