# Correction

**Published:** 1905-05

**Authors:** 


### CORRECTION.

On page 263 of the April number, in the report of the discussion of the Academy of Stomatology, Dr. M. H. Cryer is arranged as one of the speakers. This is an error, as he desires it understood that he took no part in the discussion.

